# Large Language Models in Dementia Assessment and Management: A Systematic Review

**DOI:** 10.1192/bjo.2026.11144

**Published:** 2026-06-30

**Authors:** Ami Mehta, Sushruth Vasuki Ramesh, Ishaan Dev, Federica Pace, Judith Harrison

**Affiliations:** 1 School of Medicine, Newcastle University, Newcastle upon Tyne, United Kingdom; 2 School of Medicine, University of Manchester, Manchester, United Kingdom; 3 Translational and Clinical Neurosciences, Biomedical Research Building Campus for Ageing and Vitality, Newcastle University, Newcastle upon Tyne, United Kingdom

## Abstract

**Aims:**

Large language models (LLMs) are increasingly proposed to support healthcare delivery, yet their ability to address the distinctive challenges of dementia care remains unclear. Dementia is characterised by progressive cognitive impairment, communication barriers, high caregiver burden, and substantial demands on memory services and old age psychiatry. We systematically reviewed and classified empirical applications of LLMs in dementia assessment and care, and critically appraised the maturity of evidence, intended users, and reporting of safety, fairness, and governance relevant to clinical implementation.

**Methods:**

We searched EMBASE, MEDLINE, PsycINFO, PubMed, the ACL Anthology, the ACM Digital Library, arXiv, medRxiv, and bioRxiv (2017–June 2025). Eligible studies were empirical evaluations (simulation-based or experimental) of LLMs used for dementia-relevant clinical, caregiver-facing, or research tasks; non-evaluative commentaries were excluded. Titles/abstracts were screened by multiple reviewers, with full-text screeningconducted in accordance with PRISMA; disagreements were resolved by discussion. Using a standardised extraction form, we recorded study design, model attributes, input modalities, dementia-related use cases, evaluation setting (benchmark/vignette vs clinical data), outcomes, and reporting of ethics approval, bias assessment, and hallucination-related issues. Findings were synthesised narratively and grouped using a predefined taxonomy of clinical, patient-facing, and research-oriented applications.

**Results:**

We identified 35 dementia-focused studies, several covering multiple applications. The most frequently reported functions were screening/early detection (26/35) and diagnostic support (28/35), typically framed as classification tasks. Other common applications included research facilitation (19/35), evaluation/outcome measurement (19/35), information extraction (14/35), and prediction/risk stratification (13/35). Multimodal approaches were reported in (14/35). Intended users were most often researchers (22/35) and clinicians (20/35), with fewer studies targeting people living with dementia (10/35) or caregivers. Inputs included speech/audio (20/35) and free text (18/35); outputs most commonly included classification labels (26/35) and risk scores (11/35). Performance metrics were reported in 18/35. Reporting of safeguards was inconsistent: ethics approval (10/35, 29%), bias assessment (10/35, 29%), and explicit hallucination reporting (5/35, 14%).

**Conclusion:**

LLMs show promising experimental performance for dementia-related classification and early detection tasks, suggesting potential roles in augmenting assessment workflows. However, evidence is largely preclinical, with limited real-world validation and inconsistent reporting of fairness, safety, and governance. Future research should prioritise prospective evaluation in memory services, robust bias and hallucination assessment, and co-designed implementation with people living with dementia and caregivers to support safe, equitable integration in psychiatric practice.

